# Ezrin is essential for the entry of Japanese encephalitis virus into the human brain microvascular endothelial cells

**DOI:** 10.1080/22221751.2020.1757388

**Published:** 2020-06-15

**Authors:** Yan-Gang Liu, Yang Chen, Xiaohang Wang, Ping Zhao, Yongzhe Zhu, Zhongtian Qi

**Affiliations:** a Department of Microbiology, Shanghai Key Laboratory of Medical Biodefense, Naval Medical University (Second Military Medical University), Shanghai, People’s Republic of China; b College of Basic Medicine, Naval Medical University (Second Military Medical University Shanghai), Shanghai, People’s Republic of China

**Keywords:** Brain microvascular endothelial cells, caveolin-1, ezrin, Japanese encephalitis virus, Src, viral entry

## Abstract

Japanese encephalitis virus (JEV) remains the predominant cause of viral encephalitis worldwide. It reaches the central nervous system upon crossing the blood–brain barrier through pathogenic mechanisms that are not completely understood. Here, using a high-throughput siRNA screening assay combined with verification experiments, we found that JEV enters the primary human brain microvascular endot*helial cells (HBMEC) through a caveolae-mediated endocytic pathway. The role of* ezrin, an essential host factor for JEV entry based on our screening, in caveolae-mediated JEV internalization was investigated. We observed that JEV internalization in HBMEC is largely dependent on ezrin-mediated actin cytoskeleton polymerization. Moreover, Src, a protein predicted by a STRING database search, was found to be required in JEV entry. By a variety of pharmacological inhibition and immunoprecipitation assays, we found that Src, ezrin, and caveolin-1 were sequentially activated and formed a complex during JEV infection. A combination of *in vitro* kinase assay and subcellular analysis demonstrated that ezrin is essential for Src-caveolin-1 interactions. *In vivo*, both Src and ezrin inhibitors protected ICR suckling mice against JEV-induced mortality and diminished mouse brain viral load. Therefore, JEV entry into HBMEC requires the activation of the Src-ezrin-caveolin-1 signalling axis, which provides potential targets for restricting JEV infection.

## Introduction

Japanese encephalitis virus (JEV) is a mosquito-borne virus that causes at least 60,000 annual cases of viral encephalitis worldwide [1]. It is most prevalent in Asia, the western Pacific, and northern Australia and is currently maintained by mosquito populations in these regions [2]. Twenty to thirty percent of JEV encephalitis cases result in death, while 30–50% of survivors sustain neurologic or psychiatric sequelae, which imposes a heavy burden on public health [3]. Although vaccines have been developed against JEV infection, there are currently no approved therapeutics for JEV-associated neurological symptoms due to poor understanding of the mechanisms underlying JEV neurotropism, blood–brain barrier (BBB) penetration, and neuroinflammation [4].

JEV reaches the brain by crossing the BBB through the hematogenous route [5]. The BBB is a physical and physiological barrier composed of tightly linked brain microvascular endothelial cells (BMEC), pericytes, astrocytes, and microglia [6]. JEV has been proposed to penetrate the BBB in two ways: (i) directly: by infecting BMEC, pericytes, and astrocytes, and reaching the CNS upon BBB disruption [5,7,8]; and (ii) indirectly: using peripheral immune cells, such as macrophages, as “Trojan horses” to carry them and to invade the BBB [9]. The BBB is generally not permissive to infiltration by peripheral immune cells; therefore, a “Trojan horse” mechanism requires increased permeability of the BBB [5]. A growing number of evidence suggests that BBB disruption occurs only after JEV infection of cells in the BBB [7,8,10]. Thus, a “Trojan horse” mechanism may be only secondary to infection of BMEC, microglia, or astrocytes. Indeed, infection and replication in BMEC have been widely accepted as the initial events of JEV penetration of the BBB [7,11–13]. Electron-microscopic analysis of the infected mouse brain indicated that JEV is transported across the BBB through caveolae-like endocytosis in BMEC [14]. Therefore, exploring the mechanism that JEV employs to infect BMEC is important in understanding how JEV penetrates the BBB.

Infection begins with viral entry, which involves a repertoire of cellular processes and hundreds of host proteins. Although some viruses have the capacity to directly cross the plasma membrane, most utilize endocytic organelles to reach intracellular compartments [15,16]. The classical clathrin-mediated endocytosis in JEV infection has been reported for multiple cell types, such as human cervical carcinoma (HeLa) [17], human hepatocarcinoma (Huh7) [18], and hamster kidney-derived BHK-21 cells [19]. More recently, we have demonstrated that JEV is internalized in neuron-derived rat neuroblastoma B104 cells and human neuroblastoma SK-N-SH cells through a caveolin-1-dependent pathway [20,21]. Consistent with these findings, Kalia et al. reported that JEV infects fibroblasts in a clathrin-dependent manner but enter neuronal cells through a clathrin-independent endocytic route [22]. However, the entry mechanism of JEV into BMECs is still poorly characterized.

Here, using a high-throughput siRNA screening assay, we tried to discover the host factors involved in JEV infect primary human brain microvascular endothelial cells (HBMEC) and to characterize the mechanism of the virus internalization in the cells. This approach may pave the way for understanding the process in JEV crossing BBB and facilitate the development of antiviral-drug.

## Materials and methods

### Cells, virus, and mice

HeLa (ATCC CCL-2), BHK-21 (ATCC, CCL-10) and SK-N-SH (ATCC HTB-11) were maintained in a Dulbecco’s Modified Eagle Medium (DMEM; Thermo Fisher Scientific, MA, USA) supplemented with 10% foetal bovine serum (FBS) (GIBCO, NY, USA) at 37°C (humidified environment, 5% CO_2_). Primary HBMECs were purchased from ScienCell Research Laboratories (CA, USA) and cultured in Endothelial Cell Medium (ECM) (ScienCell). Experiments were conducted using recently thawed cells at passage 6–16. JEV strain SA14 (GenBank accession no. U14163.1) was propagated in BHK-21 cells, and viral titres were determined in BHK-21 cells by plaque formation assay. One-day-old suckling mice (ICR strain) were used for this study. All animal experiments were approved by the Laboratory Animal Ethics Committee at the Naval Medical University, Shanghai, China, and performed in accordance with the approved guidelines.

### RNA interference screening

RNA interference screening was performed as previously described [23]. Briefly, HBMEC (2×10^4^ cells/well) were seeded in collagen-coated 96-well black optical-bottom plates (Nunc, Thermo Fisher Scientific). Cells (70% confluence) were transfected with 75 nM siRNAs and incubated for 60 h. After infection with JEV at a multiplicity of infection (MOI) of 10 for 48 h, the cells were fixed and then subjected to immunofluorescence staining with rabbit anti-JEV-NS3 antibody. Collation and analysis of image data were performed as previously described [23]. The nuclei were stained with 4’, 6’-diamidino-2-phenylindole (DAPI, Roche, Basel, Switzerland) and counted to determine total cell populations, while AF-488-stained cytoplasm were counted to determine the number of virus-infected cells. The Z’ score was determined as previously described to ensure minimal signal variation [24]. SiRNAs that resulted in ≥50% reduction in the number of stained JEV-infected cells in duplicate screens were regarded as positive candidates JEV suppression. Three independent screening assays were performed.

### Viral endocytosis kinetics assay (internalization assay)

HBMEC were grown on 24-well plates (1×10^5^ cells/well) and pre-treated with JEV (MOI of 50) in 500 μl ECM (4°C, 1 h) two days after confluence. The cells were washed with PBS, supplied with fresh ECM, and transferred to 37°C. At the indicated times, washed cells were treated with or without proteinase K (1 mg/mL) (Thermo Fisher Scientific) for 30 min at 4°C to remove adsorbed but not internalized virus. After washing three times with PBS, total RNA was isolated, and JEV RNA copy numbers were detected by qRT-PCR. For each timepoint, the copy numbers of JEV RNA from cells treated with proteinase K represent intracellular virus content (2^−ΔΔC*t*−I^), while those from cells treated with PBS instead of proteinase K represent total virus content (2^−ΔΔC*t*−*t*^). The JEV RNA copy number in the PBS control group at 0 h (2^−ΔΔC*t*−*t*0^) was set as 100%. Therefore, the copy numbers of the internalized virus were expressed as follows:

$$\mathrm{Internalized}\;\mathrm{virus}=2^{-\Delta \Delta \mathrm{Ct}-I}-(2^{-\Delta \Delta \mathrm{Ct}-t}-2^{-\Delta \Delta \mathrm{Ct}-t0})$$

### In vitro kinase assay

*In vitro* kinase assay was performed as described previously [25]. In brief, Escherichia coli expressed GST fusion proteins (GST-ezrin, GST-caveolin-1) were purified by GSTrap FF Columns (GE Healthcare, IL, USA), cleaved for GST portion by thrombin (Sigma Aldrich), and further purified by HiTrap Benzamiidine FF Columns (GE Healthcare) according to the manufacturer’s instructions. 2μg of these polypeptides were incubated with commercial Src (active, 0.5 μg, Abcam) and ATP (20 μM, Cell Signaling Technology) in kinase buffer (Cell Signaling Technology), with or without PP2 (1 μM), for 30 min at 37°C. The reaction was stopped by adding SDS-PAGE loading buffer and boiling. Phosphorylated ezrin or caveolin-1 was detected by western blotting.

### Immunoprecipitation

Immunoprecipitation was performed as previously described [25]. Briefly, 5 μg of rabbit anti-Src (2108S), anti-caveolin-1, and anti-ezrin antibodies or rabbit IgG isotype control were incubated overnight at 4 °C by gentle rotation (14 rpm) with 1 mL lysates (prepared with lysis buffer containing 50 mM Tris-HCl, 150 mM NaCl, 1% Nonidet P-40, 0.1% NaN_3_, 0.5% deoxycholate, 0.1% SDS, 1 mM orthovanadate, and protease/phosphorylase inhibitor mixture; pH 7.4) from JEV-exposed or PBS-treated HBMEC, and then incubated with 50 μl pre-washed Protein G Agarose (Roche-Sigma Aldrich) for 3 h at 4 °C. The samples were washed four times with lysate buffer, eluted by mixing with LDS sample buffer and boiled at 100 °C for 10 min. The samples were analysed by immunoblotting with appropriate antibodies using ECL detection reagents.

### Quantifying G-/F-Actin ratio

G-actin and F-actin were extracted and analysed as previously described [26]. HBMEC grown in 60-mm dishes, followed by lysis and centrifugation as previously described. To de-polymerize F-actin, the precipitate was resuspended with an equal volume of actin destabilization buffer (0.1 M PIPES, 1 mM MgSO4, 10 mM CaCl2, 5 μM cytochalasin D). Equal volumes of the fractions were subjected to beta-actin analysis by western blotting with β-actin antibody.

### Virus infection and administration of inhibitors in mice

Mice in the same nest were divided randomly into three groups and injected subcutaneously (sc.) with 0.02 mL (4 × 10^3^ PFU) JEV followed by PBS (0.02 mL), NSC668394 (6 mg/kg), or PP2 (7.5 mg/kg) sc., respectively. The drugs (include PBS) were continued daily for 5 days. The survival of mice was monitored for 14 days after JEV inoculation. In another experiment under similar treatment, on 5th-day post-infection, alive mice in each group were sacrificed. Brain from each mouse was halved sagittally, half was lysed with TRIzol for RT–PCR, and the other half was fixed with 4% paraformaldehyde for immunostaining. The stained brain slides were scanned by Pannoramic MIDI (3D HISTECH, Budapest, Hungary) and analysed by Pannoramic scanner (3D HISTECH).

Additional materials and methods are listed in Supplementary Materials and Methods.

### Statistical analysis

Data are expressed as the arithmetic mean ± standard deviation (SD) of at least three independent experiments. Statistical difference between group means were evaluated by one-way analysis of variance (ANOVA), followed by Bonferroni post-hoc analysis using GraphPad Prism 7.00 (GraphPad Software, CA, USA). *P* < 0.05 was considered statistically significant.

## Results

### Identification of membrane-trafficking factors involved in JEV infection by siRNA screening

**Figure 1.**
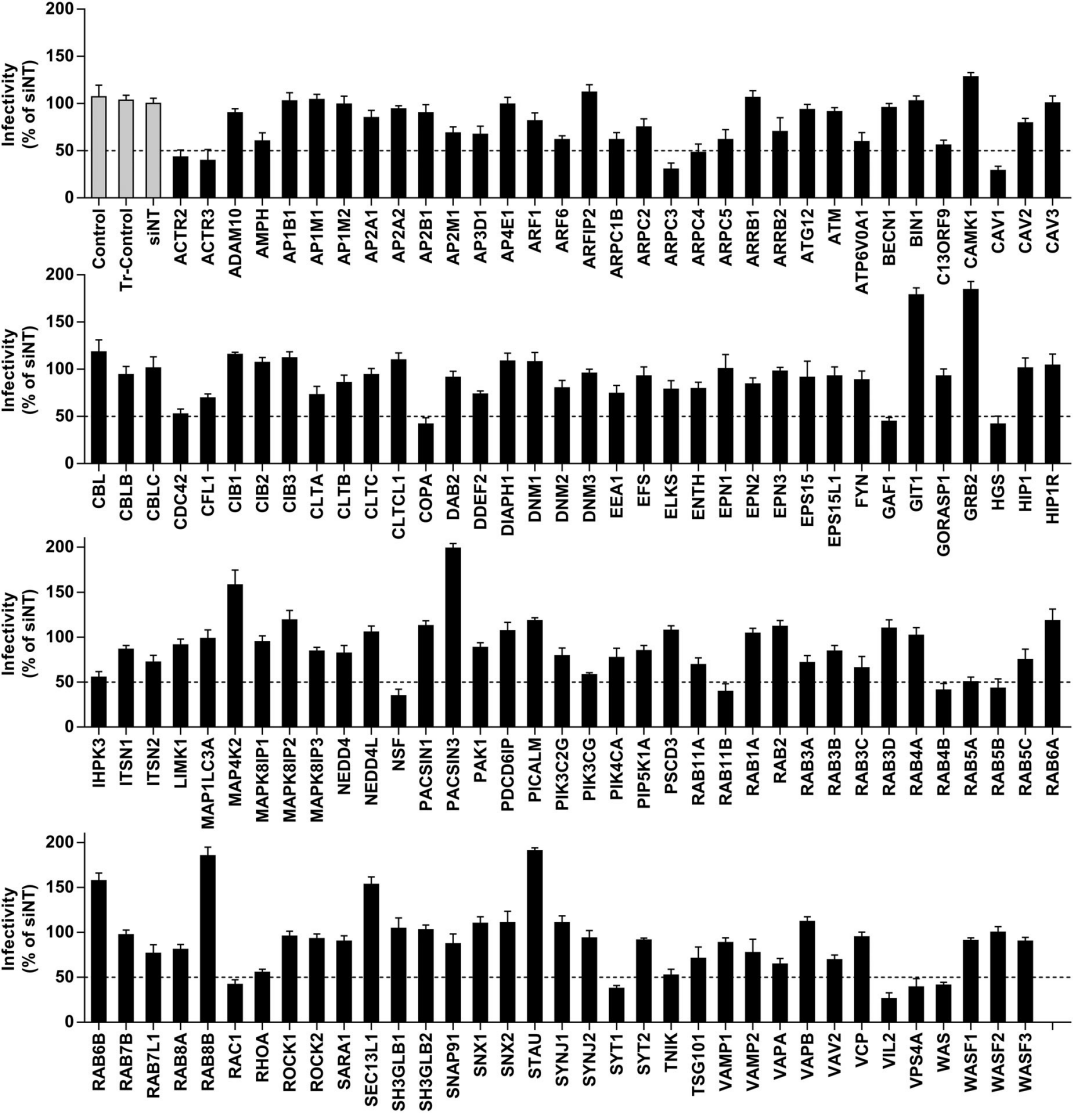
Identification of membrane-trafficking factors involved in JEV infection. HBMEC were transfected with Human Membrane Trafficking siRNA library and infected with JEV (MOI of 10). Immunofluorescence assays were performed to detect JEV. The percentage of viral antigen-positive cells were calculated and normalized to siNT (non-targeting siRNA). Genes with 50% decrease in JEV infectivity (dotted line) compared to the siNT control were considered for further analysis. Transfection control: Tr-control; vehicle control: control.

To identify cellular factors involved in JEV infection in HBMEC cells, we utilized a robust single-round, high-throughput siRNA screening assay. SiRNAs that reduced the number of infected cells by ≥50% relative to the number of infected cells with non-targeting siRNA (siNT) were regarded as candidates for JEV inhibition. As shown in Figure 1, 18 out of 140 siRNAs were identified as possible targets for JEV inhibition. Brief descriptions of the reported functional roles and sequences of the siRNAs for each of these genes is provided in Table S1. Cell viability was measured, and minimal cytotoxicity was observed after transfection (data not shown). The genes that scored positive were then classified into several protein classes. SiRNAs targeting caveolin-1 (*CAV1*), the principal component of caveolae membranes and involved in receptor-independent endocytosis, displayed a significant inhibitory effect on JEV infection. Other targets for reducing JEV infectivity were genes encoding the ESCRT machinery (*HGS* and *VPS4A*); genes for mediating membrane fusion (*NSF* and *SYT1*); genes involved in actin polymerization (*ACTR2*, *ACTR3, ARPC3, ARPC4, RAC1, VIL2*, and *WAS*); and genes involved in vesicle and endosomal transport (*COPA*, *GAF1*, *RAB4B*, *RAB5A*, *RAB5B*, *and RAB11B*).

### JEV entry into HBMEC via a caveolae-mediated endocytic pathway

**Figure 2.**
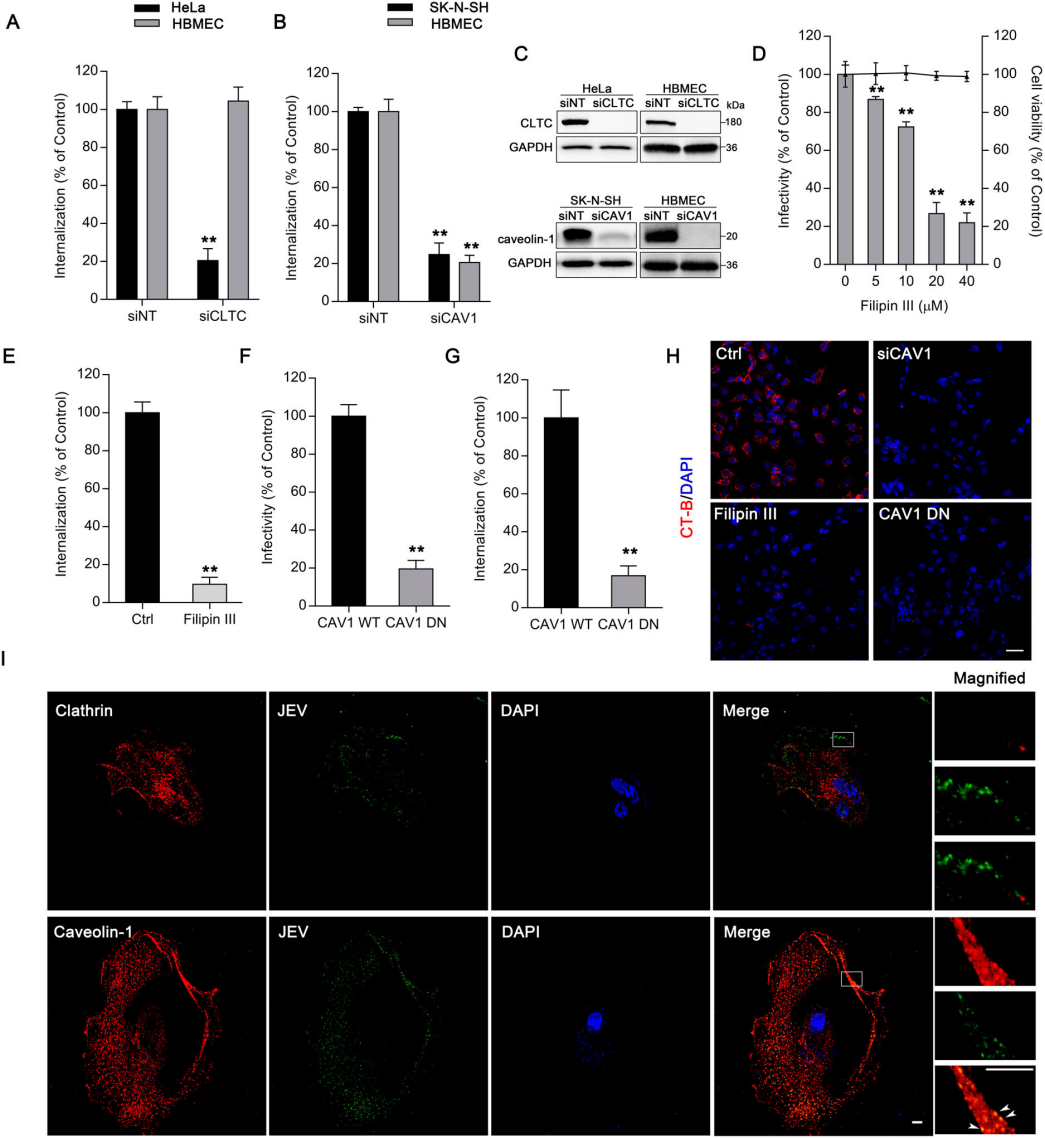
JEV entry into HBMEC via a caveolae-mediated endocytic pathway. Cells transfected with (A) siCLTC or (B) siCAV1 were incubated with JEV (MOI of 50), and internalization assays were performed. (C) Protein levels of clathrin heavy chain (CLTC) and caveolin-1 were measured by western blotting with GAPDH as reference. Values are normalized against that of non-targeting siRNA (siNT) control (A, B, C). (D) HBMEC were pre-treated with different concentrations of filipin III and infected with JEV (MOI of 10). JEV infectivity was measured by immunofluorescence. Cell viability was tested by the CCK-8 kit. (E) HBMEC were with and without (control) filipin III (20 μM) treatment were incubated with JEV (MOI of 50), and internalization assay was performed. Values were normalized against that of DMSO control (D, E). (F, G) Cells expressing wild-type (WT) or dominant-negative (DN) forms of caveolin-1 were infected with JEV (MOI of 10 or 50). Infectivity was analysed by immunofluorescence; intracellular JEV RNA, by internalization assay. Values are normalized against that of WT control. (H) AF-555 CT-B was added to cells pre-treated with filipin III, or transfected with siCAV1, or expressing caveolin-1 DN. The cells were examined by confocal microscopy. Scale bar, 200 μm. (I) Confocal microscopy localization of clathrin or caveolin-1 (red) with JEV (MOI of 100) (green). Arrowheads: JEV and caveolin-1 colocalization. Nuclei were DAPI-stained. Insets show magnified boxed areas. Scale bars, 20 μm. Results are representative of three independent experiments. **p < 0.01 compared to control.

Our RNAi screen indicated that silencing of *CAV1*, but not of factors involved in clathrin-mediated pathways (*AP1B1*, *AP2A1*, *CLTA*, *CLTB*, *CLTC, ENTH,* and *EPN2*), resulted in a significant reduction in JEV infectivity (29.08% of siNT group), although JEV infectivity (73.05%) with siRNA against *CLTA* was statistically different from that with siNT (Figure 1). To determine the role of clathrin and caveolin-1 in JEV internalization, an endocytic assay was performed 2 h after viral entry, based on the viral entry kinetic curve (Figure S1). Depletion of clathrin heavy chain by siRNA (siCLTC) did not affect JEV entry in HBMEC (Figure 2(A)). In the control, siCLTC inhibited JEV entry into HeLa cells, which have been previously reported to internalize JEV through the clathrin-dependent endocytic pathway [17]. Pre-treatment of HBMEC with CPZ, an inhibitor of clathrin recycling and clathrin-coated vesicle formation, did not exhibit obvious inhibitory effects on JEV infection and uptake (Figure S2A and S2B). Therefore, we presumed that clathrin-mediated internalization is not required for JEV infection in HBMEC.

We next investigated whether caveolin-mediated endocytosis is involved in JEV infection. Silencing of *CAV1* significantly reduced JEV entry into HBMEC (Figure 2(B)), similar to the case in SK-N-SH cells, which we have previously reported to employ caveolae-mediated internalization [21]. Interference efficiency of siRNAs were tested with immunoblotting (Figure 2(C)). Pre-treatment with filipin III, which inhibits caveolin-mediated endocytosis, inhibited JEV infection in a dose-dependent manner (Figure 2(D)). JEV internalization was also markedly interrupted by 20 μM filipin III (Figure 2(E)). The expression of dominant-negative mutants of caveolin-1 (*CAV1* DN) also had reduced JEV infectivity and entry (Figure 2(F,G)). CT-B internalization, usually used as a marker of caveolae-dependent endocytosis, was inhibited by siCAV1, filipin III, and *CAV1* DN in HBMEC (Figure 2(H)). Double-staining for clathrin or caveolin-1 and JEV of infected HBEC revealed that JEV is widely colocalized with caveolin-1 2 h p.i., but not with clathrin, at the membrane ruffles and cytoplasm (Figure 2(I)). These data suggest that JEV entry in HBMEC is caveolin-dependent.

### Ezrin-mediated actin reorganization is essential for JEV entry

As shown in the screening (Figure 1), the ezrin-targeted siRNA resulted in the lowest JEV infectivity (26.24% of siNT) out of all the candidates (the interference efficiency of the siRNA was tested with immunoblotting, Figure S3A). Ezrin is a member of ERM protein family that consists of two more closely related proteins, i.e. moesin and radixin [27]. ERM proteins are tether proteins that are crucial in supplying functional linkages between membrane proteins and the cytoskeleton to regulate membrane protein dynamics and cytoskeleton rearrangement [27]. Recent studies suggest that other viruses modulate pathways involving ezrin to ensure viral entry [28-30]. However, its role in JEV infection is not known.

**Figure 3.**
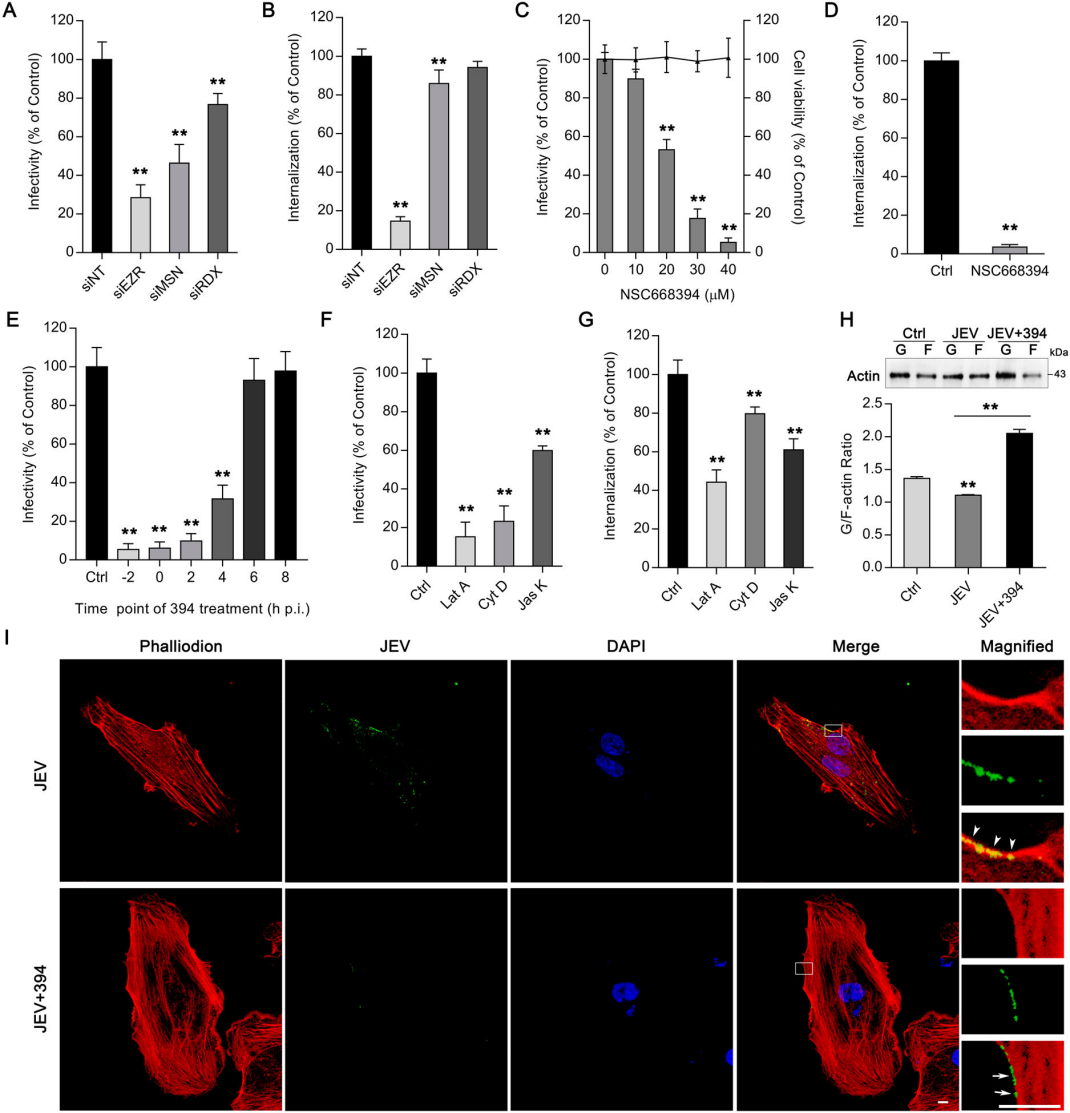
Ezrin-mediated actin reorganization is essential for JEV entry. HBMEC were transfected with siRNAs targeting ERM protein family members (EZR, MSN, RDX). (A) JEV (MOI of 10) infectivity and (B) intracellular JEV RNA (initial MOI of 50) were determined. Values were normalized against that of non-targeting siRNA (siNT) control. (C) HBMEC were treated with ezrin inhibitor NSC668394 at different concentrations and infected with JEV (MOI of 10) for 2 h. JEV infectivity was measured by immunofluorescence. Cell viability was tested by CCK-8 kit. (D) HBMEC were treated with and without (control) NSC668394 (30 μM) and incubated with JEV (MOI of 50); internalization assay was performed to analyse intracellular JEV RNA. (E) HBMEC bound with JEV (MOI of 10; 4°C, 2 h) were transferred to 37°C. At the indicated times (h.p.i, hours post-infection), cells were treated with NSC668394 (30 μM). Forty-eight hours after transfer to 37°C, JEV infectivity was analysed by immunofluorescence (−2: simultaneous addition of NSC668394 and JEV). (F,G) Cells were pre-treated with latrunculin A (Lat A, 5 μM), cytochalasin D (Cyt D, 10 μM), or jasplakinolide (Jas K, 5 μM), then infected with JEV (MOI of 10 or 50). JEV infectivity (F) and JEV internalization (G) were determined. Values were normalized against that of DMSO control (Ctrl; C-G). (G) Western blotting analysis shows fractions of G-actin and F-actin in JEV-infected HBMEC infected (MOI of 50) with or without NSC668394 (30 μM). The G-actin/F-actin ratio is shown below. Cells treated with DMSO were used as solvent control. (H) Cells exposed to JEV (MOI of 100) with or without NSC668394 (30 μM) for 2 h were stained with tetramethylrhodamine-conjugated phalloidin (red) and anti-JEV-NS3 (green) and observed by confocal microscopy. Arrowheads: colocalization of JEV and actin at membrane dorsal ruffles; arrows: non-overlapping JEV and actin on membrane. Nuclei were DAPI-stained. Insets show magnified boxed areas. Scale bars, 20 μm. Results are representative of three independent experiments. **p < 0.01 compared to control or indicated group (lined).

Therefore, we next explored the roles of ERM proteins (ezrin, moesin, and radixin) in JEV infection of HBMEC. Knockdown of these proteins reduced JEV infectivity, but only ezrin depletion exhibited marked decrease in JEV entry (Figure 3(A,B)), although the moesin knockdown resulted in significantly reduced internalization (85.83% of the siNT group). Treatment with NSC668394, a cell-permeable ezrin phosphorylation-specific inhibitor, reduced JEV infection in a dose-dependent manner (Figure 3(C)). Internalization of JEV was also markedly disrupted by 40 μM of NSC668394 (Figure 3(D)). JEV infection was significantly inhibited when NSC668394 was added at −2, 0, 2, and 4 h p.i., whereas the addition of NSC668394 at 6 h p.i. or at later time points had the least inhibitory effects on viral infection (Figure 3(E)). These indicate that ezrin plays an essential role in JEV internalization in HBMEC.

We next investigated the role of ezrin in regulating actin cytoskeleton dynamics during JEV infection. The impact of actin polymerization or depolymerization perturbation on virus internalization was confirmed by treatment with three cell-permeant compounds, latrunculin A, jasplakinolide, and cytochalasin D. All the three compounds especially latrunculin A, a depolymerizing agent, showed significant interference with JEV infectivity and internalization (Figure 3(F,G)). As a measure of actin polymerization, we quantified the G/F-actin ratio of JEV-infected cells, with or without NSC668394 (40 μM) treatment (Figure 3(H)). Results showed that the G/F-actin ratio declined upon JEV infection, suggesting that actin polymerization was induced, and that this was reversed by NSC668394 treatment. Furthermore, subcellular localization analysis of actin cytoskeleton and JEV particles by double staining showed that HBMEC exhibited profound actin cytoskeleton reorganization and formation of membrane ruffles, which largely colocalized with viral particles (arrowheads, Figure 3(I)). Notably, these were markedly inhibited by treatment with NSC668394 (arrows, Figure 3(I)). Taken together, these data indicate that ezrin-mediated cytoskeleton remodelling is involved in JEV entry into HBMEC.

### Ezrin is critical for Src/caveolin-1 interaction during JEV entry

**Figure 4.**
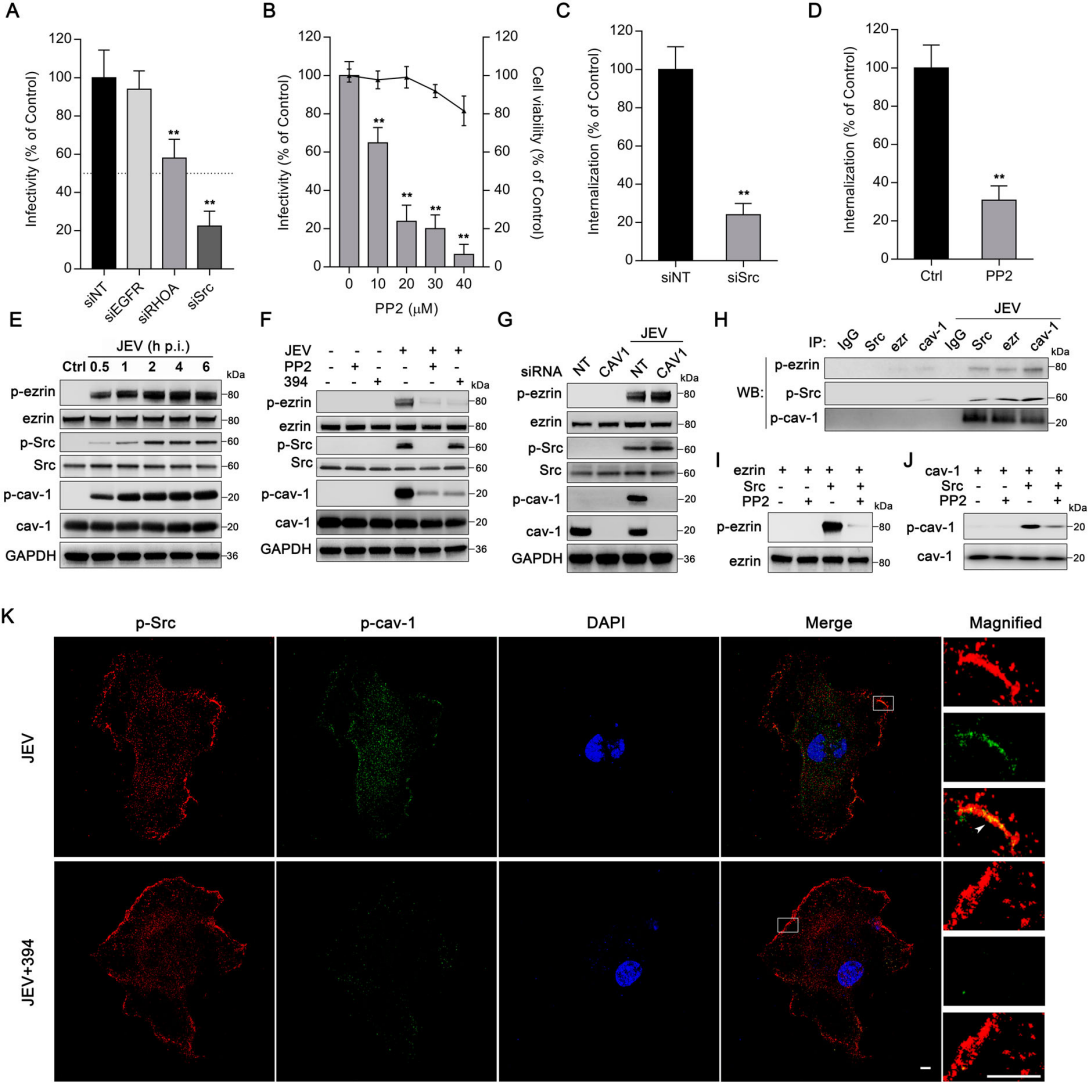
Ezrin is critical for Src/caveolin-1 interaction during JEV entry. HBMEC (A) transfected with siRNA targeting EGFR, RhoA, or Src, or (B) treated with PP2 at different concentrations were infected with JEV (MOI of 10). JEV infectivity was assessed by immunofluorescence. Cell viability was tested by CCK-8 kit. Dotted line: 50% JEV infectivity compared to the siNT control. HBMEC were (C) transfected with siSrc or (D) treated with PP2 (20 μM) and incubated with JEV (MOI of 50) and JEV internalization was determined. Values are normalized against that of non-targeting siRNA (siNT) or DMSO control (Ctrl) (A–D). (E–G) Western blot analyses of HBMEC cell lysates. (E) HBMEC were infected with JEV (MOI of 50) for 0.5, 1, 2, 4, 6 and blotted using an anti-p-ezrin, anti-ezrin, anti-p-Src, anti-Src, anti-p-caveolin-1 (p-cav-1), anti-caveolin-1 (cav-1), and anti-GAPDH (internal reference) antibodies. Ctrl: PBS control. (F) HBMEC were treated with NSC668394 (30 μM) or PP2 (20?M) and infected with JEV (MOI of 50) for 2 h. (G) HBMEC transfected with non-targeting siRNA (siNT) or siCAV1 were infected with JEV (MOI of 50) for 2 h. (H) Lysates from JEV-infected (MOI of 50) or PBS-treated HBMEC were subjected to immunoprecipitation (IP) with antibodies against Src, ezrin (ezr) or caveolin-1 (cav-1), or non-specific IgG controls. Immunoprecipitates were analysed by western blotting (WB) for the presence of phosphorylated proteins. (I, J) In vitro kinase assays: recombinant (I) ezrin, or (J) caveolin-1 was untreated, phosphorylated with recombinant Src or phosphorylated with Src, and further dephosphorylated with PP2 (1 μM). Half-reactions were analysed by western blotting using anti-p-ezrin or anti-p-caveolin-1 antibody; the other half were analysed using anti-ezrin or anti-caveolin-1 antibody. (K) Cells treated with DMSO control or NSC668394 (30 μM) were exposed to JEV (MOI of 100) for 2 h, stained with p-Src (red) and p-caveolin-1 (p-cav-1, green), and observed by confocal microscopy. Arrowheads: Src and caveolin-1 colocalization at the membrane ruffle. Nuclei: DAPI-stained. Insets show magnified boxed areas. Scale bars, 20 μm. Results are representative of three independent experiments. **p < 0.01 compared to control.

We then utilized the database Search Tool for the Retrieval of Interacting Genes (STRING, http://string-db.org/, version 11.0) [31] to infer proteins associated with both ezrin and caveolin-1. We found the epidermal growth factor receptor (EGFR), Src, and the ras homolog family member A (RhoA) as directly correlative candidates (Figure S4). Using siRNA and specific inhibitors showed that interference of Src but not of EGFR nor Rho A led to significant reduction (>50%) in JEV infectivity (Figure 4(A,B) and Figure S5). Even though siRHOA and CTO4 (a Rho inhibitor) also showed statistical difference compare to siNT group, their inhibition efficiencies are both lower than 50% (Figure 4(A) and Figure S5). Src depletion by siRNA (siSrc) or inhibition by PP2 (20 μM) significantly restricted JEV internalization (Figure 4(C,D)), thereby supporting the role of Src activation in JEV entry and transduction into HBMEC. The interference efficiency of siSrc was tested with immunoblotting (Figure S3B).

The physiological Src proto-oncogene is a non-receptor tyrosine kinase that phosphorylates diverse substrates, including ezrin and caveolin-1 [25,32,33]. Phosphorylated caveolin-1 (p-caveolin-1) was reported to in turn initiate the formation of endocytic carriers during JEV infection [21]. To elucidate the correlation of Src, ezrin, and caveolin-1, their activation was investigated at 0.5, 1, 2, 4, 6 h post-JEV infection. Immunoblotting for phosphorylation showed that Src, ezrin, and caveolin-1 were all activated 30 min after JEV infection, and the levels of activated proteins increased and were maintained during JEV entry (Figure 4(E)). Treatment of cells with PP2 (20 μM) appeared to block JEV-activated Src, ezrin, and caveolin-1, while NSC668394 only inhibited the activation of ezrin and caveolin-1 but not of Src (Figure 4(F)). Additionally, depletion of caveolin-1 by siRNA even enhanced the activation of Src and ezrin (Figure 4(G)). Of note, JEV or inhibitors of Src and ezrin resulted in minimal influence on the expression levels of the proteins (Figure 4(E,F)). Therefore, the results strongly suggest that JEV infection-induced Src/ezrin/caveolin-1 sequential activation and that caveolin-1 plays a role in the negative feedback of Src/ezrin activation. As ezrin and caveolin-1 have been reported to be substrates of Src [25,34], coimmunoprecipitation assays and *in vitro* kinase assays were then performed to examine the interaction between Src and ezrin or caveolin-1. Immunoprecipitation of Src, ezrin or caveolin-1, after JEV infection, all led to the isolation of p-Src, p-ezrin and p-caveolin-1(Figure 4(H)). As a control, none of the interaction partners were co-precipitated with rabbit IgG isotype control. These results indicate that activated Src, ezrin, and caveolin-1 form a supramolecular complex during JEV invasion. The results of i*n vitro* kinase assays showed that Src can phosphorylate both ezrin and caveolin-1 *in vitro* (Figure 4(I,J)). Immunostaining of p-Src and p-caveolin-1 two hours after JEV infection, with or without NSC668394 treatment, showed that the JEV induced colocalization of p-Src and p-caveolin-1 on the membrane (arrowhead, Figure 4(K)) was blocked by NSC668394, with a significant reduction of caveolin-1 activation and no change in Src activation (Figure 4(K)). It appears that ezrin, which was activated by Src, was required for directly phosphorylation of caveolin-1 by Src during JEV infection.

### Inhibition of ezrin or Src protects mice from JEV infection-induced lethality in vivo

**Figure 5.**
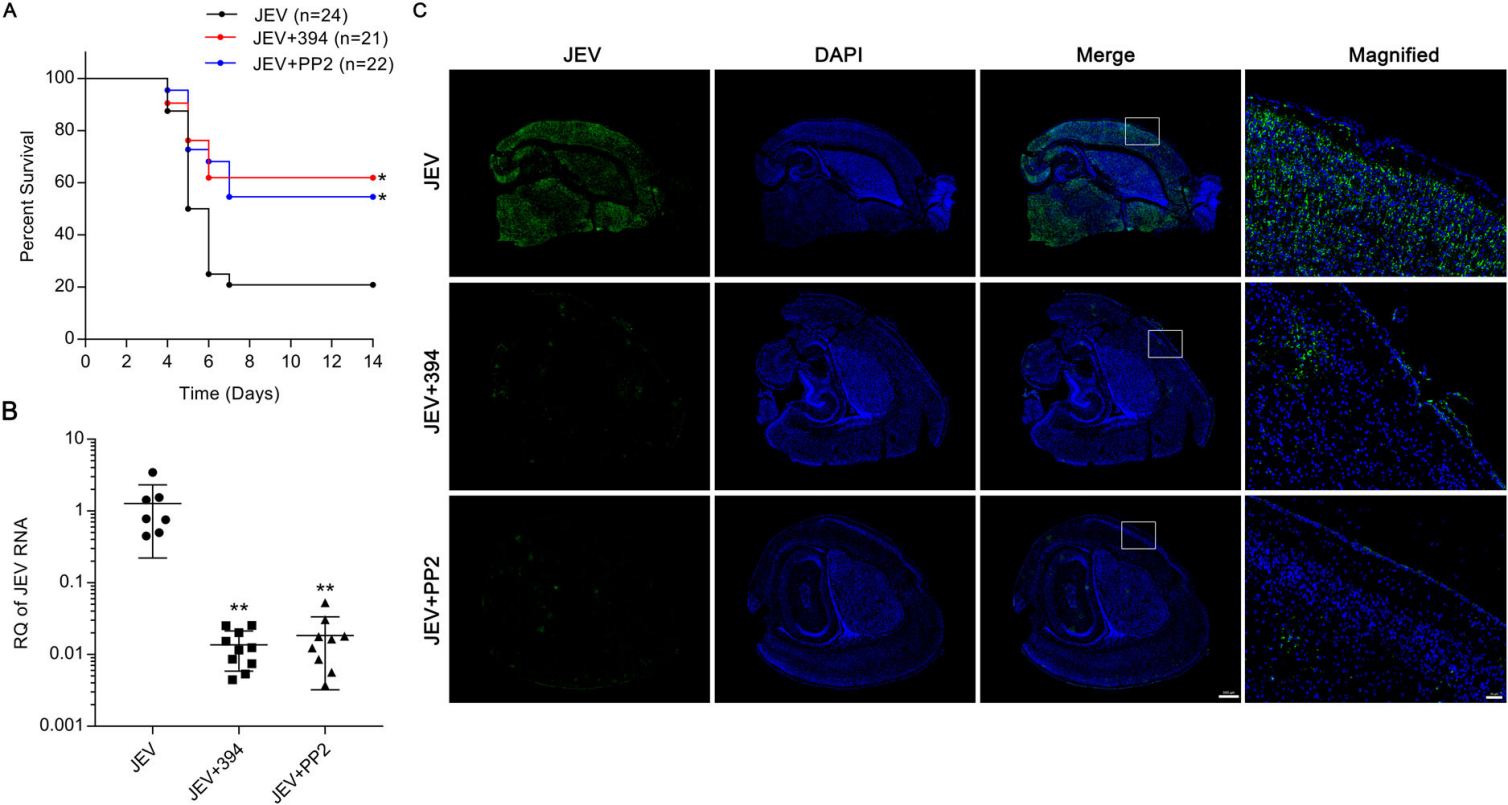
Inhibition of ezrin or Src protects mice from JEV infection-induced lethality. Mice were infected with JEV (4×103 PFU/mouse) and treated with PBS, NSC668394 (6 mg/kg) or PP2 (7.5 mg/kg) through the subcutaneous route. The drugs (include PBS) were continued daily for 5 days. (A) Kaplan-Meier survival curves for 14 days post-infection are shown. (B) JEV RNA in brain was quantified by qRT-PCR assay 5 days post-infection. Results are denoted as the relative quantity (RQ) of JEV RNA genome compared to the JEV (PBS) group. JEV, *n* = 7; JEV+394, *n* = 10; JEV + PP2, *n *= 9. (C) Mouse brain sections were stained for JEV-NS3 protein (green); nuclei were stained with DAPI. Insets show magnified boxed areas. Scale bars in scanned and magnified images are 500 and 50 μm, respectively. Results are representative of three independent experiments. *p < 0.05 compared to the JEV-infected, PBS-treated group. **p < 0.01 compared to the JEV-infected, PBS-treated group.

To assess the possibility of targeting Src and ezrin for inhibiting JEV infection, we examined the protective effects of Src (PP2) or ezrin (NSC668394) inhibitors in mice challenged with JEV. First, to determine if NSC668394 or PP2 could rescue JEV-induced mouse death, suckling ICR mice-infected s.c. with a lethal dose of JEV were administered either PBS or PP2 or NSC668394 and monitored daily for survival. Mice in the JEV + PBS group started to show symptoms, including whole-body tremor and limb paralysis, 3 days p.i. From 4 to 7 days p.i., 79.2% (19 out of 24) mice in the JEV-infected group succumbed to the infection. Administration of PP2 and NSC668394 both significantly relieving symptoms and reduced mortality (54.5% and 61.9% survival, respectively; Figure 5(A)). Assessment of viral titres in mouse brain indicate that, in line with survival analysis, repeated administration of PP2 and NSC668394 both significantly reduced viral load in the brain, compared to the PBS-treated group (Figure 5(B)). Immunofluorescence assay for JEV-NS3 antigen in mouse brains showed that JEV is widely present in PBS-administered mouse brain slides and was dramatically diminished in PP2- and NSC668394-treated mice. Therefore, inhibitors of ezrin or Src protected mice from JEV-induced lethality, showing that Src or ezrin are potential anti-JEV drug targets.

## Discussion

Infection of BMEC is a crucial first step for JEV penetration into the BBB, but the JEV entry mechanism into BMEC is currently poorly understood [7,11,13]. Here, we report the identification of ezrin as an essential factor for Src-caveolin-1 interaction that facilitates Src-mediated caveolin-1 phosphorylation, leading to JEV internalization. This mechanism for JEV infection of BMEC has not been reported so far, and it provides potential targets for the development of antivirals.

Through RNA-interference-based screening, we were able to characterize a previously unreported role for ezrin in JEV infection of HBMEC. Recently, by RNAi screening, Khasa et al. have shown that genes related to the clathrin-mediated endocytosis were involved in JEV infection of HeLa cells; however, ezrin was excluded [17]. Although we have previously reported that JEV entry in neurons is caveolae-dependent, we also did not detect the involvement of ezrin [21]. Therefore, ezrin plays a completely distinct role in HBMEC in JEV uptake. Ezrin is a structural organizer of membrane proteins and the underlying actin cytoskeleton [35]. It shows various levels of activation, depending on the signal [35] and exists in different isoforms, function, and cellular localization in different cell types [36]. JEV, which infects a broad range of cell types [37], may use different routes when adhering to BMECs and to HeLa cells, which may explain differences in ezrin utility in these cells. In polarized cells, ezrin is concentrated at the apical domain, characterized by microvilli [36], which might also be the case in cells of the brain microvascular endothelium, which are also heavily polarized [38].The distribution of ezrin to the microvilli in BMEC may bridge the gap between membrane proteins to facilitate signalling during JEV infection. This may also explain why JEV stimulation activates Src although JEV entry does not involve ezrin in neuronal cells, as reported by our group and by Das et al. [21,39].

The mechanisms of caveolin-mediated endocytosis remain largely uncharacterized. In this study, we observed the successive activation of Src, ezrin, and caveolin-1 and the organization of p-Src/p-ezrin/p-caveolin complex during JEV infection in HBMEC. Interestingly, by *in vitro* kinase assay, we noted that activated Src was able to directly phosphorylate ezrin and caveolin-1. However, the activation of caveolin-1 was blocked by treatment with a specific inhibitor of ezrin phosphorylation without a change in p-Src, indicating a key role for ezrin in mediating Src-caveolin-1 interaction in JEV infection. Since caveolin-1 is located in lipid raft domains while Src is in non-raft membrane regions in the resting cell [39,40], a scaffold or process to promote their interaction may be required. In addition to its structural role, ezrin has been reported to function in organizing receptor proteins and intracellular proteins, thereby orchestrating their signal transduction [41]. After activation, ezrin in an open conformation was capable of targeting protein kinase A (PKA) to downstream substrates in lipid rafts by interaction with raft transmembrane proteins [42]. Pidoux et al. reported that, after activation by PKA, ezrin directs PKA to a molecular complex and acts as a scaffold for their reaction [43]. Therefore, in JEV infection, it is possible that after activation by Src, ezrin directs and anchors Src to caveolin-1-enriched lipid raft regions and acts as a platform for caveolin-1 phosphorylation by Src.

**Figure 6.**
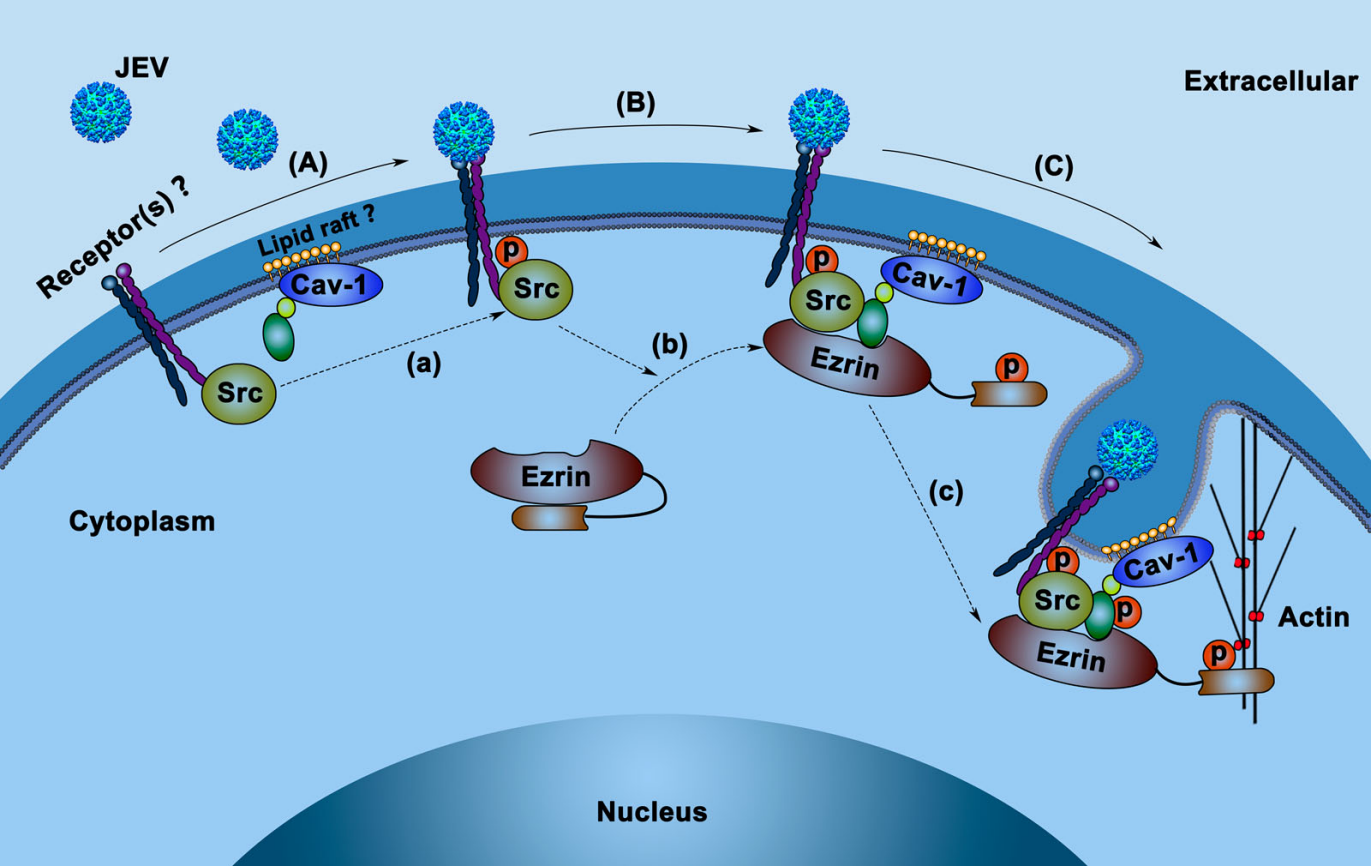
Proposed model of JEV entry route upon HBMEC infection. (A) JEV attaches to HBMEC surface receptors (e.g. integrins), co-receptors, or other attachment proteins, leading to the phosphorylation of Src (a). (B) Phosphorylated-Src induces phosphorylation and activation of ezrin (b). Ezrin in its open conformation (p-ezrin) anchors p-Src to the caveolin-1-enriched region (probably a lipid raft). (C) p-Src induces the phosphorylation and activation of caveolin-1 on the scaffold of p-ezrin (c) and mediates the formation of endocytic carriers in synergy with ezrin-mediated actin polymerization.

Interestingly, cells knockout of caveolin-1 showed a higher level of activated Src (p-Src) and ezrin (p-ezrin) than the control group under JEV infection (Figure 4(G)). As ezrin and caveolin-1 were reported to be substrates of Src [25,34] (this was also verified in our study, Figure 4(I,J)), this suggested that p-Src can phosphorylate caveolin-1, and caveolin-1 activation may possess a feedback inhibition to Src activation with unknown mechanism. Indeed, a caveolin-1-Src negative feedback regulation loop has been characterized in studies [44-46]. Chen et al. reported that activation of caveolin-1 negatively feedback Src activation via inhibiting endothelial nitric oxide synthase (eNOS) phosphorylation in endothelial (Figure 6 in the paper) [45]. Cao et al. demonstrated that phosphorylation of caveolin-1 can leads to C-terminal Src kinase (Csk) translocation into caveolae which may also induce a feedback loop that result in inactivation of the Src family kinases [46].

In this study, inhibitors of Src (PP2) and ezrin (NSC668394) have exhibited conspicuous therapeutic effects on JEV infection in a suckling ICR mouse model by dramatically reducing viral loads in brain and improving mouse survival rate. A previous study has shown that PP2 has protective effects against JEV-induced neurotoxicity in cultured neurons and glia, but its anti-JEV activity *in vivo* was not investigated [47]. Although we could not rule out the possibility that the viral load in circulation was diminished by the inhibitors before entry into the BBB, our results still provide promising pharmacological targets in JEV infection. However, a specific inhibitor for calveolin-1 is not yet available, so we could not test whether it could similarly protect mice.

We also noticed that the interference of RhoA by siRNA and inhibitor CT04 both restrained the infectivity of JEV. Although their inhibition efficiencies are both lower than 50% (Figure 4(A) and Figure S5), it did indicate a role of RhoA in JEV infection in HBMEC. A possible explanation may be that there is a compensatory mechanism for maintaining JEV infection after suppression of RhoA. Additionally, our current study did not address the functional role of endosomal transportation- or membrane fusion-related proteins detected in the screen, which may also be possible antiviral targets.

Our results suggest a model explain signalling transduction for initiating JEV entry into BMEC where ezrin directs Src to caveolin (Figure 6), where ezrin acts as an anchor for Src-caveolin-1 interaction to promote caveolin-1 activation by Src. Taken together, using a primary HBMEC culture model, we report for the first time that ezrin organizes a supramolecular signalling complex and plays an essential role in JEV entry in BMEC. Moreover, we show that Src and ezrin are promising targets for the development of therapeutics against JEV.

## Supplementary Material

Supplemental Material
