# Supplementary material for: Ezrin is essential for the entry of Japanese encephalitis virus into the human brain microvascular endothelial cells
Source: Emerg Microbes Infect. 2020 Jun 15;9(1):1330–41. doi: 10.1080/22221751.2020.1757388 (PMC7473060; doi:10.1080/22221751.2020.1757388)
Supplement: Supplemental Material [file TEMI_A_1757388_SM0387.docx]

**Ezrin is essential for the entry of Japanese encephalitis virus into the human brain microvascular endothelial cells**

Yangang Liu^a,1^, Yang Chen^a,b,1^, Xiaohang Wang ^a,b,1^, Ping Zhao^a^, Yongzhe Zhu^a,*^, and Zhongtian Qi^a,*^

^a^ Department of Microbiology, Shanghai Key Laboratory of Medical Biodefense, Naval Medical University (Second Military Medical University), 200433 Shanghai, China. ^b^ College of Basic Medicine, Naval Medical University (Second Military Medical University Shanghai), 200433 Shanghai, China.

^1^ These authors contribute equally to this work.

* Correspondence: Zhong-Tian Qi (qizt@smmu.edu.cn) or Yong-Zhe Zhu (zhuyongzhe1984@sina.com), Department of Microbiology, Shanghai Key Laboratory of Medical Biodefense, Naval Medical University (Second Military Medical University Shanghai), No. 800, Xiangyin Road, 200433 Shanghai, China, Tel: +86 218 187 0988

Running Title: Essential role of ezrin in JEV entry

**Supplementary** **Materials and Methods**

## Reagents

Rabbit anti-JEV-NS3 (GTX125868) antibody, mouse anti-JEV-E (GTX40735) antibody, and rabbit IgG isotype control were purchased from GeneTex (TX, USA). Rabbit anti-Src (ab47405), anti-phosphorylated ezrin (ab76247), anti-clathrin heavy chain (ab21679), and anti-caveolin-1 (ab2910) antibodies were purchased from Abcam (Cambridge, England). Rabbit anti-Src (2108S), anti-ezrin (3145S), and anti-GAPDH (2118S) antibodies were purchased from Cell Signaling Technology (MA, USA). Mouse anti-phosphorylated c-Src antibody was purchased from Santa Cruz (sc-166860; TX, USA). Mouse anti-phosphorylated caveolin-1 antibody (MAB7418) was purchased from R&D Systems (MN, USA). Rabbit anti-actin antibody was purchased from ABclonal Technology (Wuhan, China). Rabbit anti-phosphorylated Src antibody (44-660G), AF-488/555-conjugated donkey anti-rabbit immunoglobulin G (IgG), Alex Fluor 488/555 (AF-488/555)-conjugated donkey anti-mouse IgG, horseradish peroxidase (HRP)-conjugated goat anti-rabbit IgG, and HRP-conjugated goat anti-mouse IgG were purchased from Thermo Fisher Scientific. Chemical inhibitors used in the drug inhibition assays include: chlorpromazine (CPZ), NSC-668394, cytochalasin D, jasplakinolide, and latrunculin A (Sigma-Aldrich, MO, USA); filipin III (Santa Cruz); PP2 and erlotinib (MedChemExpress, NJ, USA); and CT04 (Cytoskeleton, CO, USA). These drugs did not affect the nature of JEV and its ability to attach (data not shown). The specificities of the inhibitors are listed in Table S2. Specific siRNAs targeting moesin, radixin, epidermal growth factor receptor (EGFR), RhoA, and Src were synthesized by GenePharma (Shanghai, China). The Human Membrane Trafficking siRNA library (G-005505, 140 targets) was purchased from Dharmacon-Thermo Fisher Scientific. Plasmid constructs expressing GFP-tagged wild-type (WT) and dominant negative (DN) mutants of caveolin-1 were kindly provided by J. M. Bergelson (University of Pennsylvania).

### Immunofluorescence

HBMEC were grown on 8-well chamber slides (Thermo Fisher Scientific). After treatment, cells were washed twice with phosphate-buffered saline (PBS), fixed with 4% paraformaldehyde for 20 min at room temperature (RT), permeabilized with 0.1% Triton X-100 (5 min), and blocked 2 h with 3% bovine serum albumin (BSA; 2 h). After incubation with primary antibodies (overnight, 4°C) and fluorescent secondary antibodies or tetramethylrhodamine-conjugated phalloidin (AAT Bioquest, CA, USA) (1 h, RT), the nuclei were counterstained with DAPI. Images were captured with an Olympus IX 81 fluorescence microscope (Olympus, Tokyo, Japan), or with Zeiss LSM 710 (Carl Zeiss, NY, USA) confocal microscope. Images were adjusted for brightness and contrast, and figures were assembled with Photoshop CC 2017 (Adobe, CA, USA).

### Western blotting

After treatment, standard western blotting was performed. Cells were lysed with radioimmunoprecipitation assay buffer (Thermo Fisher Scientific) followed by sonication (1 min) and boiling (10 min). Samples were normalized to equal concentrations of protein, separated on 10% sodium dodecyl sulphate-polyacrylamide gel electrophoresis (SDS-PAGE), and transferred onto polyvinylidene fluoride (PVDF) membranes (Bio-Rad, CA, USA). Membranes were blocked with 5% skimmed milk, incubated overnight (4°C) with the indicated primary antibodies. After washing with Tris-buffered saline (TBS)-Tween 20 (0.1%), membranes were incubated with secondary HRP-conjugated antibodies (2 h), followed by washing. Results were visualized with the enhanced chemiluminescence (ECL Plus) western blotting detection reagents (PerkinElmer Life Sciences, MA, USA).

### Quantitative real time PCR (qRT-PCR)

Total RNA was extracted from treated HBMEC or mouse brains using TRIzol (TaKaRa, Shiga, Japan). First strand cDNA synthesis was performed using PrimeScript RT Master Mix (TaKaRa) according to manufacturer’s instructions. PCR amplification of JEV RNA and glyceraldehyde 3-phosphate (*GAPDH*; reference gene) was performed with SYBR Premix Ex Taq (TaKaRa) using respective primer pairs (Table S3) in an ABI 7300 system (Applied Biosystems, MA, USA). Copy numbers of JEV RNA were normalized against *GAPDH* levels using comparative cycle threshold values (2^-ΔΔCt^) determined in parallel.

### Cholera Toxin Subunit B (CT-B) uptake assay

HBMEC were grown on 8-well chamber slides. Forty-eight hours after confluence, AF-555 CT-B (C34776; Molecular Probes, OR, USA) was added for 2 h and removed by washing with 0.1 M glycine, 150 mM NaCl (pH 2.5) prior to fixation. After three washes with PBS, the samples were fixed with methanol (-20℃, 20 min), washed with PBS, and viewed using Zeiss LSM 710 confocal microscope.

### Cell viability assay

Cell viability was assessed using a CCK-8 cell counting kit (Vazyme Biotech, Nanjing, China) following manufacturer’s instructions. Cells were seeded in a 96-well plate (2×10^4^ cells/well) and allowed to reach full confluence for 48 h. Following treatment with drugs (2 h) or siRNAs (60 h), the cells were washed twice with PBS. CCK-8 solution diluted in ECM (ScienCell) was then added, followed by incubation for 2 h. Absorption at 450 nm was determined using a microplate reader (BioTek, Vermont, USA). The percentage of viable cells was calculated using the formula (OD, optical density):

ratio (%) = [OD_Treatment_ – OD_Blank_] / [OD_Control_ – OD_Blank_]×100

### Cell transfection and transient expression

For plasmid overexpression, cell transfection was conducted according to manufacturer’s instructions for Lipofectamine 3000 (Thermo Fisher Scientific). Briefly, HBMEC were seeded in collagen-coated 24-well tissue culture plates (5×10^4^ cells/well). When the cells have reached 70% confluence, the DNA-lipid complexes were prepared as follows: 0.8 μg plasmid in 50 μl of Opti-MEM (Thermo Fisher Scientific) and 50 μl of Opti-MEM with 1 μl of P3000 reagent and 2 μl of Lipofectamine 3000; incubated for 20 min. After incubation, the cell supernatant was changed to 400 μl fresh ECM, and the freshly prepared DNA-lipid complexes were added to the cells. The mixture was incubated for 48 h.

**Supplementary Figures and Tables**

**
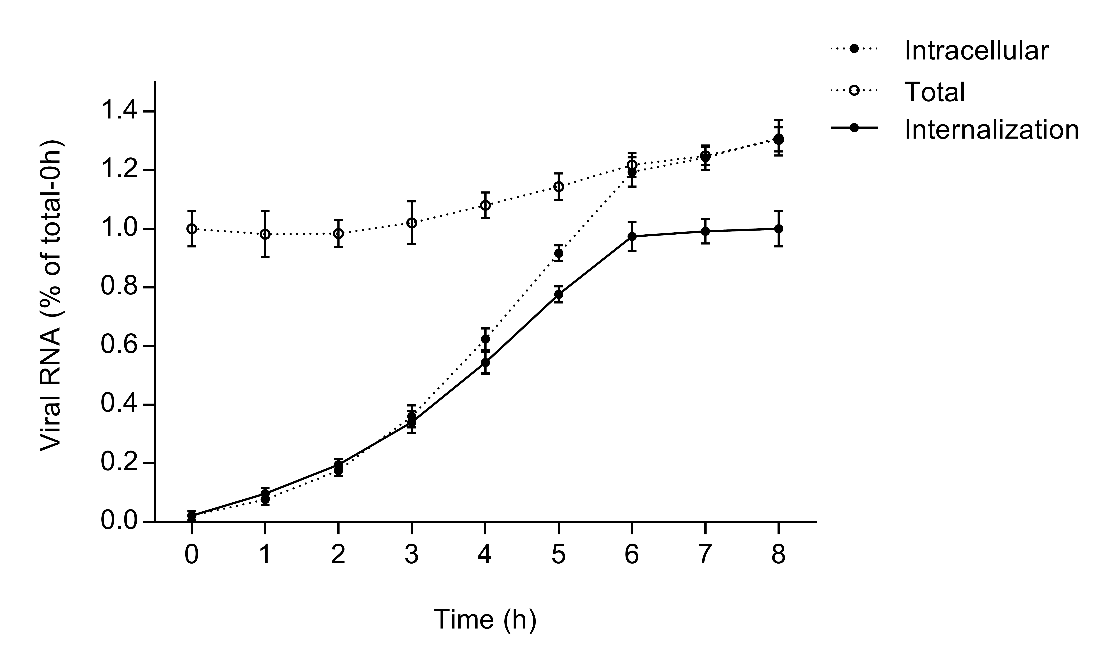
**

**Figure S1. Kinetics of JEV entry into** **HBMEC.**

HBMEC were pre-treated with JEV (MOI of 50) at 4 °C for 1 h and transferred to 37 °C. At the indicated times, cells were treated with or without proteinase K. Total RNAs were isolated, and the copy numbers of JEV RNA were detected by qRT-PCR analysis. The total (group without proteinase K) JEV RNA is shown by the dotted line with empty circles. The intracellular JEV RNA is shown by the dotted line with filled circles. The internalized JEV RNA calculated based on the two lines is exhibited as a solid line.


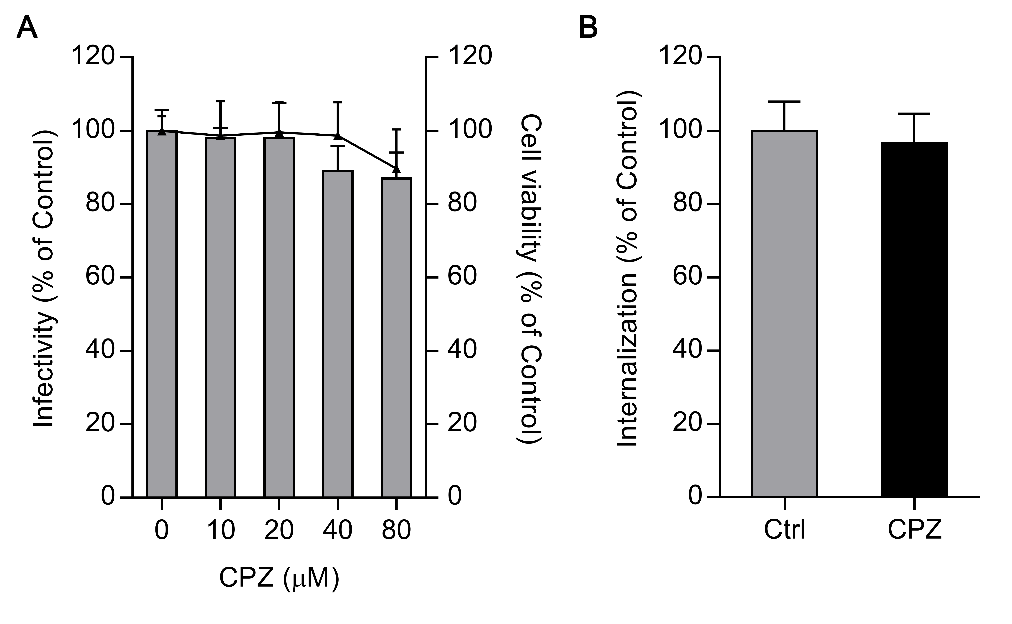


**Fig S2.** **JEV infection of HBMEC does not involve clathrin.**

**(A)** HBMEC were pre-treated with chlorpromazine (CPZ) at different concentrations then infected with JEV (MOI of 10). JEV infectivity was measured by immunofluorescence. Cell viability was tested by CCK-8 kit. **(B)** HBMEC were left untreated (control) or were treated with CPZ (40 μM), and then incubated with JEV (MOI of 50). Internalization assay was performed to determine intracellular JEV RNA. Values are normalized against that of the DMSO control.


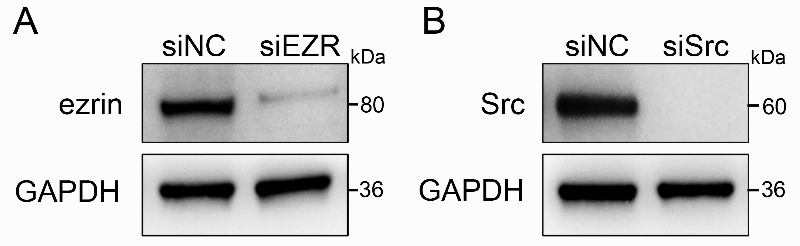


**Fig S3. Analysis of the interference efficiency of siRNAs.**

HBMEC were transfected with (**A**) siEZR or (**B**) siSrc and protein levels of ezrin and Src were measured by western blotting with GAPDH as reference. kDa: molecular weight.


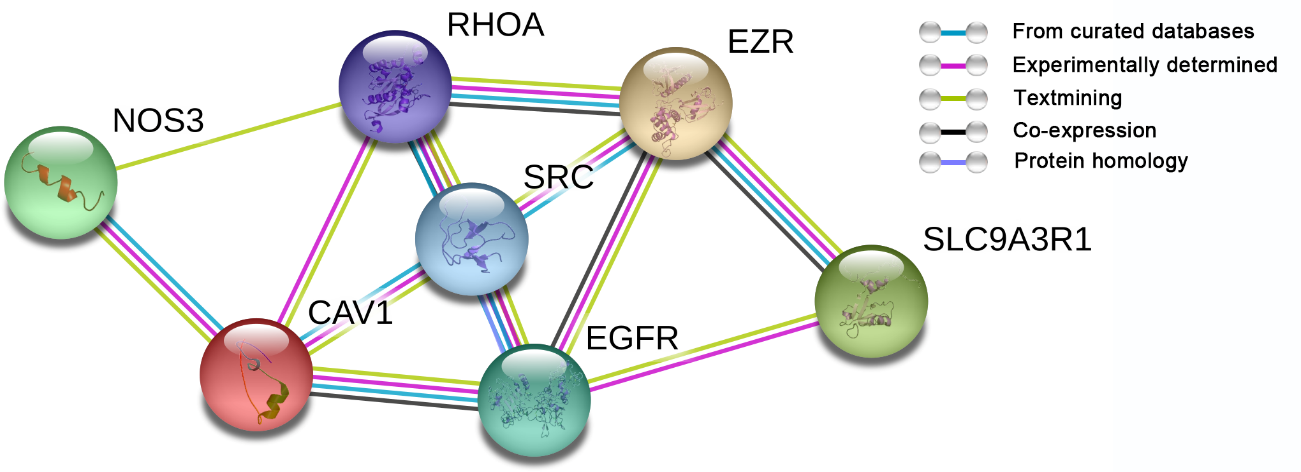


**Fig S4. Protein-protein interaction network produced using STRING.**

STRING 11.0 was utilized to predict proteins that interact with both ezrin and caveolin-1. Colored nodes indicate the individual protein identified. The number of lines represent strength of predicted functional interactions between proteins. STRING, Search Tool for the Retrieval of Interacting Genes/Proteins. NOS3: nitric oxide synthase 3. CAV1: caveolin-1. RHOA: ras homolog family member A. EGFR: epidermal growth factor receptor. EZR: ezrin. SLC9A3R1: solute carrier family 9 member A3 regulator 1.


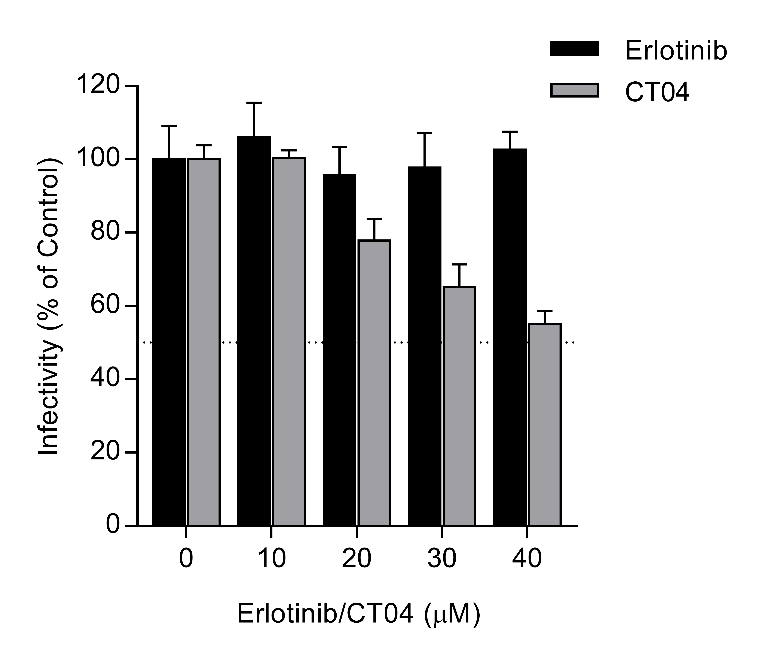


**Fig S5.** **JEV infection of HBMEC is independent of EGFR and RhoA.**

HBMEC pre-treated with different concentrations of erlotinib, and CT04, were infected with JEV for 48 h. Immunofluorescence was utilized to assess the infectivity of JEV. Values were normalized against that of the DMSO control. The dotted line indicates 50% of JEV infectivity relative to the siNT control group.

**Table S1. Genes involved in JEV infection of HBMEC**

| **Genes** | **Gene Accession** | **Name and Function of Genes** | **siRNA Sequence (5’-3’)**  **(D_x_: Duplex Number)** |
| --- | --- | --- | --- |
| ACTR2 | NM_005722 | ARP2 actin-related protein 2 homolog, a major constituent of the ARP2/3 complex, which is essential to cell shape and motility through lamellipodial actin assembly and protrusion | D_1_: GAAGUUAACUACCCUAUGG  D_2_: GCAAGUGAAUUACGAUCAA  D_3_: GAAACGGUUCGCAUGAUUA  D_4_: UGGUGUGACUGUUCGAUAA |
| ACTR3 | NM_005721 | ARP3 actin-related protein 3 homolog, a major constituent of the ARP2/3 complex, which is essential to cell shape and motility through lamellipodial actin assembly and protrusion | D_1_: GGAAUAUACUGCUGAAAUA  D_2_: GACCUAGACUUCUUCAUUG  D_3_: GAACAAUCCUUGGAAACUG  D_4_: UAAAGGAGCGCUAUAGUUA |
| ARPC3 | NM_005719 | Actin related protein 2/3 complex, subunit 3. The Arp2/3 protein complex has been implicated in the control of actin polymerization in cells and has been conserved through evolution | D_1_: GCAAGAGACUGGACUGAGA  D_2_: GAUGAGAGCCUAUUUACAA  D_3_: AUACAGAUAUUGUGGAUGA  D_4_: CAAACAAACAGGAAGAUGA |
| ARPC4 | NM_001024960 | Actin related protein 2/3 complex, subunit 3. The Arp2/3 protein complex has been implicated in the control of actin polymerization in cells and has been conserved through evolution | D_1_: GAAGAGUUCCUUAAGAAUU  D_2_: AGAUGAAGCUGUCAGUCAA  D_3_: GAACUUCUUUAUCCUUCGA  D_4_: GGUAUGAUAUCAGCUUUCU |
| CAV1 | NM_001753 | Caveolin-1, in molecular biology caveolins are a family of integral membrane proteins that are the principal components of caveolae membranes and involved in receptor-independent endocytosis | D_1_: CUAAACACCUCAACGAUGA  D_2_: GCAGUUGUACCAUGCAUUA  D_3_: AUUAAGAGCUUCCUGAUUG  D_4_: GCAAAUACGUAGACUCGGA |
| COPA | NM_004371 | Coatomer protein complex, subunit alpha, one subunit of COP I complex, mediates proteins transport between the endoplasmic reticulum and Golgi compartments | D_1_: ACUCAGAUCUGGUGUAAUA  D_2_: GCAAUAUGCUACACUAUGU  D_3_: GAUCAGACCAUCCGAGUGU  D_4_: GAGUUGAUCCUCAGCAAUU |
| GAF1 | NM_015470 | RAB11 family interacting protein 5 (class I), which is important for protein trafficking from ARE to the apical plasma membrane | D_1_: GCAACAAGCUGCGCAAGUC  D_2_: GGUACAAGCUGCACUCCAA  D_3_: GUACGUCGGUGGUGGAGAA  D_4_: GGCCAAGAGUAGCUGGUUU |
| HGS | NM_004712 | Hepatocyte growth factor-regulated tyrosine kinase substrate, which regulates endosomal sorting and plays a critical role in the recycling and degradation of membrane receptors | D_1_: GCACGUCUUUCCAGAAUUC  D_2_: AGAGAGCGAUGCCAUGUUU  D_3_: GAUAUUCUGUGGAAAGUGU  D_4_: GUAAACGUCCGUAACAAGA |
| NSF | NM_006178 | N-Ethylmaleimide-Sensitive Factor, a key factor for eukaryotic trafficking, including protein and hormone secretion and neurotransmitter release | D_1_: UGGAAAUGCUUAACGCUUU  D_2_: GGUUUAGAAUGCUGCGCUU  D_3_: GUUACAUUAUGAACGGUAU  D_4_: UGGACUAAGUGGAACGUUC |
| RAB11B | NM_004218 | RAB11B, member RAS oncogene family. Members of the Rab11 subfamily act in recycling of proteins from the endosomes to the plasma membrane, in transport of molecules from the trans-Golgi network to the plasma membrane and in phagocytosis. | D_1_: CGAGUACGACUACCUAUUC  D_2_: GACAGAAGCCCAACAAGCU  D_3_: GGAUUCCACUAACGUAGAG  D_4_: UAACGUAGAGGAAGCAUUC |
| RAB4B | NM_016154 | RAB4B are members of the RAS superfamily of small GTPases that are involved in vesicular trafficking | D_1_: GCACUAUCCUCAACAAGAU  D_2_: AGAAUGAGCUGAUGUUCCU  D_3_: GGUGAUUGGCAGUGCAGGA  D_4_: UCAGUGACGCGGAGUUAUU |
| RAB5A | NM_004162 | RAB5A, member RAS oncogene family, localizes to early endosomes where it is involved in the recruitment of RAB7A and the maturation of these compartments to late endosomes. | D_1_: GCAAGCAAGUCCUAACAUU  D_2_: GGAAGAGGAGUAGACCUUA  D_3_: AGGAAUCAGUGUUGUAGUA  D_4_: GAAGAGGAGUAGACCUUAC |
| RAB5B | NM_003929 | RAB5B, member RAS oncogene family. Protein transport. Probably involved in vesicular traffic | D_1_: GAAAGUCAAGCCUGGUAUU  D_2_: GCUAUGAACGUGAAUGAUC  D_3_: CAAGCCUGGUAUUACGUUU  D_4_: ACAAACGUAUGGUGGAGUA |
| RAC1 | NM_006908 | Members of this superfamily appear to regulate a diverse array of cellular events, including cell growth, cytoskeletal reorganization, antimicrobial cytotoxicity, and the activation of protein kinases | D_1_: UAAGGAGAUUGGUGCUGUA  D_2_: UAAAGACACGAUCGAGAAA  D_3_: CGGCACCACUGUCCCAACA  D_4_: AUGAAAGUGUCACGGGUAA |
| SYT1 | NM_005639 | Synaptotagmin-1, is an integral membrane protein of synaptic vesicle thought to serve as a sensor for calcium ions (Ca2+) in the process of vesicular trafficking and exocytosis | D_1_: GCAAUUUACUUUCAAGGUA  D_2_: GUAAGAGGCUGAAGAAGAA  D_3_: GAUCGUUUCUCUAAGCAUG  D_4_: CGACUGUUCUGCCAAGCAA |
| VIL2 | NM_001111077 | Ezrin, also known as cytovillin or villin-2, can be phosphorylated by protein-tyrosine kinase and is a member of the ERM protein family. This protein serves as a linker between plasma membrane and actin cytoskeleton. It plays a key role in cell surface structure adhesion, migration, and organization | D_1_: GCUCAAAGAUAAUGCUAUG  D_2_: GGCAACAGCUGGAAACAGA  D_3_: CAAGAAGGCACCUGACUUU  D_4_: GAUCAGGUGGUAAAGACUA |
| VPS4A | NM_013245 | Vacuolar protein sorting 4 homolog A ：The protein encoded by this gene is a member of the AAA protein family, associates with the endosomal compartments, and are involved in intracellular protein trafficking | D_1_: GCUGAAGGAUUAUUUACGA  D_2_: UCAAAGAGAACCAGAGUGA  D_3_: GAAUAACAAUGAUGGGACU  D_4_: GAGCCAAGUGCGUGCAGUA |
| WAS | NM_000377 | Wiskott-Aldrich syndrome, involved in transduction of signals from receptors on the cell surface to the actin cytoskeleton. | D_1_: CGAGAACCAGCGACUCUUU  D_2_: GCUGGCCACUGCAGUUGUU  D_3_: GAACAUACCCUCCACCCUC  D_4_: CCUCUAAACUUAUCUACGA |

**Table S2. List of chemical inhibitors**

| **Inhibitor** | **Specifity** | **Concentrations/** **dosage** |
| --- | --- | --- |
| Chlorpromazine | Inhibitor of clathrin-dependent endocytosis | 10 μM, 20 μM, 40 μM, 80 μM |
| Filipin III | Disruption of caveolar | 5 μg/ml, 10 μg/ml, 20 μg/ml, 40 μg/ml |
| PP2 | Reversible Src kinases inhibitor | 10 μM, 20 μM, 30 μM, 40 μM  *In vivo*: 7.5 mg/kg·day^-1^ |
| NSC668394 | Reversible ezrin inhibitor | 10 μM, 20 μM, 30 μM, 40 μM  *In vivo*: 6 mg/kg·day^-1^ |
| Erlotinib | EGFR tyrosine kinase inhibitor | 10 μM, 20 μM, 30 μM, 40 μM |
| CT04 | Rho Inhibitor | 10 μM, 20 μM, 30 μM, 40 μM |
| Cytochalasin D | Cell-permeable and potent inhibitor of actin polymerization | 10 μM |
| Jasplakinolide | Promotes actin polymerization and stabilizes actin filaments | 5 μM |
| Latrunculin A | Reversible inhibitor of actin assembly | 5 μM |

**Table S3. Sequences of primer pairs specific to target gene**

| **Target Gene** | **Sequences of qRT-PCR Primer Pair (5’-3’)** |
| --- | --- |
| **GAPDH** | Forward Primer TGGGCTACACTGAGCACCAG  Reverse Primer AAGTGGTCGTTGAGGGCAAT |
| **JEV** | Forward Primer CCCTCAGAACCGTCTCGGAA  Reverse Primer CTATTCCCAGGTGTCAATATGCTGT |
